# Salinity Effects on Guard Cell Proteome in *Chenopodium quinoa*

**DOI:** 10.3390/ijms22010428

**Published:** 2021-01-04

**Authors:** Fatemeh Rasouli, Ali Kiani-Pouya, Lana Shabala, Leiting Li, Ayesha Tahir, Min Yu, Rainer Hedrich, Zhonghua Chen, Richard Wilson, Heng Zhang, Sergey Shabala

**Affiliations:** 1 International Research Centre for Environmental Membrane Biology, Foshan University, Foshan 528000, China; fatemeh.rasouli@utas.edu.au (F.R.); L.Shabala@utas.edu.au (L.S.); yumin0820@hotmail.com (M.Y.); 2 Tasmanian Institute of Agriculture, College of Science and Engineering, University of Tasmania, Hobart, TAS 7001, Australia; ali.kianipouya@utas.edu.au; 3 Shanghai Centre for Plant Stress Biology and CAS Centre for Excellence in Molecular Plant Sciences, Chinese Academy of Sciences, Shanghai 201602, China; ltli@psc.ac.cn; 4 Department of Biosciences, COMSATS University Islamabad, Park Road, Islamabad 45550, Pakistan; ayesha.tahir2007@gmail.com; 5 Institute for Molecular Plant Physiology and Biophysics, University Wuerzburg, 97082 Wuerzburg, Germany; hedrich@botanik.uni-wuerzburg.de; 6 School of Science and Health, Hawkesbury Institute for the Environment, Western Sydney University, Penrith, NSW 2747, Australia; Z.Chen@westernsydney.edu.au; 7 Central Science Laboratory, University of Tasmania, Hobart, TAS 7001, Australia; richard.wilson@utas.edu.au

**Keywords:** quinoa, guard cell, stomata, salt stress, proteomics analysis

## Abstract

Epidermal fragments enriched in guard cells (GCs) were isolated from the halophyte quinoa (*Chenopodium quinoa* Wild.) species, and the response at the proteome level was studied after salinity treatment of 300 mM NaCl for 3 weeks. In total, 2147 proteins were identified, of which 36% were differentially expressed in response to salinity stress in GCs. Up and downregulated proteins included signaling molecules, enzyme modulators, transcription factors and oxidoreductases. The most abundant proteins induced by salt treatment were desiccation-responsive protein 29B (50-fold), osmotin-like protein OSML13 (13-fold), polycystin-1, lipoxygenase, alpha-toxin, and triacylglycerol lipase (PLAT) domain-containing protein 3-like (eight-fold), and dehydrin early responsive to dehydration (ERD14) (eight-fold). Ten proteins related to the gene ontology term “response to ABA” were upregulated in quinoa GC; this included aspartic protease, phospholipase D and plastid-lipid-associated protein. Additionally, seven proteins in the sucrose–starch pathway were upregulated in the GC in response to salinity stress, and accumulation of tryptophan synthase and L-methionine synthase (enzymes involved in the amino acid biosynthesis) was observed. Exogenous application of sucrose and tryptophan, L-methionine resulted in reduction in stomatal aperture and conductance, which could be advantageous for plants under salt stress. Eight aspartic proteinase proteins were highly upregulated in GCs of quinoa, and exogenous application of pepstatin A (an inhibitor of aspartic proteinase) was accompanied by higher oxidative stress and extremely low stomatal aperture and conductance, suggesting a possible role of aspartic proteinase in mitigating oxidative stress induced by saline conditions.

## 1. Introduction

Photosynthesis, the most important biochemical reaction in the world, will not occur in plants unless carbon dioxide is allowed to enter the leaves through stomatal pores, the apertures of which are controlled by guard cell (GC) movements. At the same time, stomata serve as major getaways for water loss through transpiration. The stomatal pore area may be only as much as 1% of total leaf surface, but diffusion rates from the leaf could be 95% as much evaporation as from the stomata [1]. Massive amounts of water and CO_2_ are passing through stomata of plant leaves each year [2], and changes in stomatal aperture in response to environmental factors impact on the flux of both carbon dioxide and water at a global level [3].

Salinity stress is one of the most detrimental environmental stresses that affects water balance through stomatal conductance. Globally, salinity affects 20% of arable land [4] and represents a major threat to global food security and sustainability of agricultural production systems [5]. Understanding responsive adaptation strategies has a primary role in enhancing the salt tolerance of crop plants and water use efficiency [6]. The plant response to salt stress is a complex trait, regulated by many genes and different pathways [7] that help a plant restore cellular homeostasis, repair the stress damage, and ultimately determine growth rate under salt stress. Salt stress activates signaling pathways related to osmotic, ionic and oxidative stresses, detoxification and growth regulation [8].

As a highly salt tolerant plant species, halophytes have evolved mechanisms to efficiently benefit from adverse saline conditions [9]. In glycophytes, salinity stress reduces stomatal conductance causing a decline in photosynthesis and transpiration rates [6]; this reduction is much smaller in halophytes [10]. Additionally, halophytes have superior abilities to regulate stomata density and size [11].

Studies on stomatal physiology and anatomy have provided insights into understanding stomatal differentiation and function. However, most studies on the effects of environmental stimuli on stomata at the cellular level have used whole leaf samples, which consist of diverse cell types. In such heterogenous samples, it is likely that important mechanisms in response to stimuli related specifically to stomata GCs have been at least partially masked by other more abundant cell types. In recent years, single cell-type analysis has become a popular in the field of biology and plant science. For instance, studies on isolated trichome [12] and epidermal bladder cells [13] and GCs [14,15] have revealed some unique patterns in differential gene expression. However, obtaining purified specialized cells in quantities sufficient for biochemical and standard molecular approaches remains a highly challenging issue that explains the relatively small number of papers published at the single-cell level.

Quinoa (*Chenopodium quinoa*) is a semi-domesticated halophytic plant with superior abiotic stress tolerance [16,17] including salinity and drought [11,18]. Studies investigating the GC response to salt stress in halophytes have to date focused on physiological or anatomical aspects. Given that the stomatal response of halophytes to salt stress is not well understood at the molecular level [10], quinoa represents a highly valuable model plant. However, no studies to date have used global “omics”-based techniques such as proteomics for the discovery of novel adaptive mechanisms in the GCs of halophytes under saline conditions.

A previous study on functional proteomics of Arabidopsis GCs [19] resulted in identification of 1734 unique proteins. Another study compared the proteome of *Brassica napus* GCs with mesophyll cells [15] leading to the discovery of 74 proteins preferentially expressed in the GCs and 143 proteins with higher abundance in the mesophyll. Specific GC proteins related to thioredoxin signaling [20] or ATP production [14] were also identified. The response of the GC proteome to CO_2_ levels [21] and abscisic acid (ABA) [22] are to date the only published research papers using proteomics to address the GC response to environmental factors.

Our study applying proteomics to investigate the effects of salt stress on the GCs of sugar beet revealed a significant proportion of differentially expressed proteins related to different abiotic stress e.g., salinity, drought, and oxidative stresses, as well as some proteins related to biotic stress [23]. Salt stress also altered the abundances of some proteins related to signaling, cell wall modification and ATP biosynthesis, indicating the high impact of salt on GCs in sugar beet. Furthermore, high levels of some proteins related to adaptation to oxidative stress under non-saline conditions suggested some constitutively active proteins may play a role in tolerance of GCs to salt stress in the latter species. Here, we sought to identify whether similar mechanisms exist in the GCs of halophytic quinoa species, and to what extent they differed from those reported for sugar beet.

It should be commented that most studies using proteomics methods to investigate the response of GCs to environmental factors have employed the model plant Arabidopsis. To the best of our knowledge, this is the first time that an experimental study on proteomics of GCs under salinity stress is reported in halophytes. Investigation of the salt-induced proteins in quinoa GCs as a halophyte species may be instrumental in the identification of salt responsive proteins mediating halophytes adaptation to hyperosmotic saline conditions, at the GC level.

## 2. Results

In the present study, quinoa plants were treated with 300 mM NaCl for three weeks. In total, searching the acquired tandem mass spectrometry data (MS/MS) against the *Chenopodium quinoa* (Wild.) protein database identified 2147 proteins, based on two or more matching peptides (Supplemental Table S1).

Rubisco activase (XP_021757275), a lipolytic enzyme GDSL esterase/lipase (XP_021763378), and auxin binding protein ABP19 (XP_021750120) were found to be the three most abundant proteins in quinoa GCs. Rubisco activase is a chloroplastic enzyme that is required for the activation of rubisco; this enzyme is also a responder to various abiotic stresses such as heat, cold, drought and salt stresses and contributes to plant acclimation to a variety of environmental stress [24]. GDSL esterases/lipases are a subfamily of lipolytic enzymes with a wide range of substrates that confer pathogenic resistance to plants [25]—it was the second most abundant protein in quinoa GC.

It is noteworthy that multiple isoforms of peroxidase enzymes, including peroxidase 4 (XP_021732931), peroxidase 12 (XP_021771547) catalase (XP_021754464) and L-ascorbate peroxidase (XP_021745974) and aspartic protease, were also among the top 10% most abundant proteins in the quinoa GC.

### 2.1. Protein Classification in the GC

In this study, we used protein classification and gene ontology (GO) analysis as complementary tools for bioinformatic analysis of the global quinoa proteomics dataset and the differentially abundant proteins under salinity stress. The Basic Local Alignment Search Tool (BLAST) was first used to obtain the protein sequences of each accession number in GCs’ proteome. Amino acid sequences of all GC proteins were used as input in the most recent version of MapMan framework, using Mercator4 software (https://plabipd.de/portal/mercator4) [26] to obtain the functional classification of GC proteins based on homologues of well-annotated proteins such as Arabidopsis.

**Figure 1:**
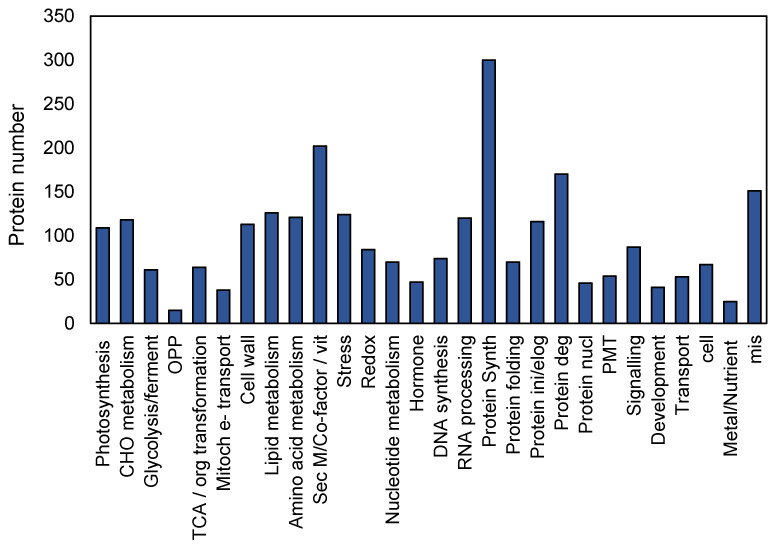
Protein functional classification of quinoa GC proteome based on the identified 2147 proteins. Amino acid sequences of all GC proteins used as input in the most recent version of MapMan framework to obtain the functional classification of GC proteins based on homologues of well-annotated proteins such as Arabidopsis.

The Mercator pipeline was used for protein functional classification analysis based on the 2147 quinoa GC proteins identified (Figure 1). This software aligns each set of protein sequence against various databases, including the Conserved Domain Database (CDD), SwissProt/Uniprot plant proteins, TAIR10 proteins, Clusters of Orthologous Groups, and then generates MapMan bin codes. Similar to Kyoto Encyclopedia of Genes and Genomes (KEGG), the MapMan framework uses massive databases spanning many pathways and functional terms but has been specifically developed for plant cell biology. The key functions identified in our dataset included protein synthesis, degradation and folding, signal transduction, post-translational modifications, biotic and abiotic stresses, development, photosynthesis, ion transporter lipid metabolism, and oxido-reductase. The eight most significantly enriched GO categories in the GC proteome are presented in Table 1. Translation, metabolic process, generation of precursor metabolites and energy were also the most significantly enriched GOs, while in most proteomic studies on whole leaf or mesophyll tissue, photosynthesis has been presented among the top GOs.

### 2.2. Signaling Proteins in Quinoa GC Proteome

The 75 signaling proteins including 14-3-3 proteins, GTP-binding proteins, mitogen-activated protein kinases, calcium-binding protein and proteins involved in light signaling were found in the GC proteome (Table 2). The 14-3-3 proteins are small acidic proteins that form homodimers and heterodimers that bind to phosphorylated target proteins and play a role in stomatal movement through the regulation of blue light responses and plasma membrane and tonoplast channels [27]. G proteins participate in several signal transduction pathways. Mutants lacking the G subunit presented hypo-sensitivity to ABA activation of anion channels and hyposensitivity to ABA inhibition of potassium channels and stomatal opening [22].

### 2.3. Transporters Proteins in Quinoa GC Proteome

Various ABC transporters from C, F, G, and I subfamilies were detected in quinoa GCs. ABC transporters (ATP-binding cassette) contribute to multiple physiological processes that lead to plant adaptation to changing environments, for example, they enhance ABA signaling, which results in a phenotype with reduced transpiration in plants under salt stress conditions [28].

V-ATPases are vacuolar H^+^ pumps that fuel tonoplast NHX (Na^+^, K^+^/H^+^) exchangers enabling sequestration of toxic Na^+^ into vacuoles, enhancing vacuolar capacity for osmoregulation and maintaining Na^+^ and K^+^ homeostasis [29]. The rate of vacuole-type ATPase activity is constitutively high in GCs compared to other cell-types to meet the requirement of rapid and large ion fluxes across the tonoplast for stomatal movements [30]. In our study, different subunits of V-type ATPase including sub-A, A3, B2, C, D, E and G were identified in quinoa GC (Table 3).

### 2.4. Differentially Abundant Proteins in Response to Salt Stress

**Figure 2:**
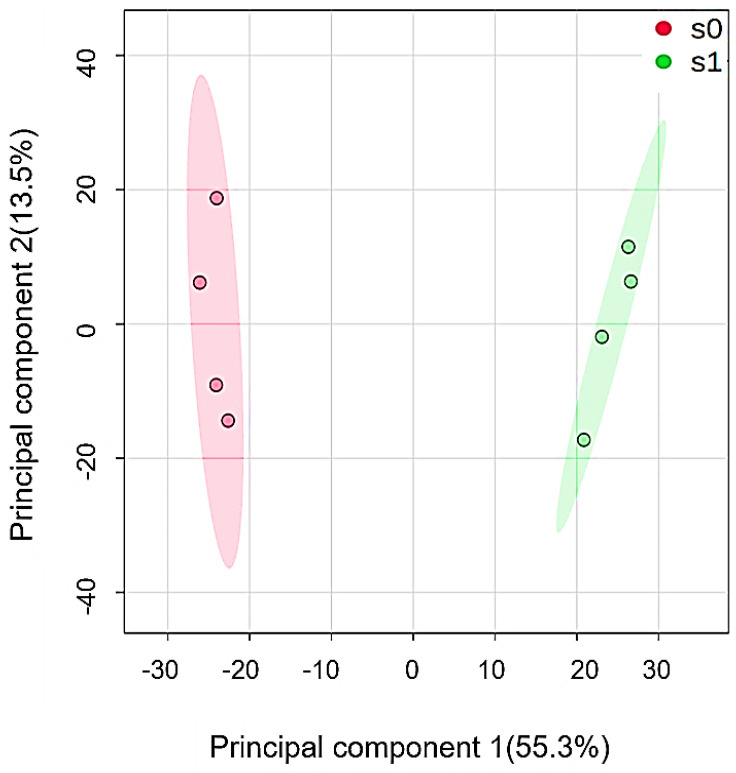
Principal component analysis (PCA) clustering based on GC proteome data under control and salt conditions. Proteomics analyses were performed with 4 replicates. S0 and S1 denote control and saline treatments, respectively.

The GC proteome data were subjected to principal component analysis (PCA) as presented in the two-dimensional biplot to find out whether the GC proteome profiles of control and salt-treated plants differ from each other (Figure 2). In the PCA plot, PC1 accounts for the difference between the salt-treated and control treatments and PC2 shows differences between biological replicates. As shown in Figure 2, PC1 explained 55.3% of the variance in the data, while PC2 captured the slight variance in protein expression profiles of GC. Hence, the separation of unstressed control and salt treatments into non-overlapping clusters suggests major differences between experimental treatments.

**Figure 3:**
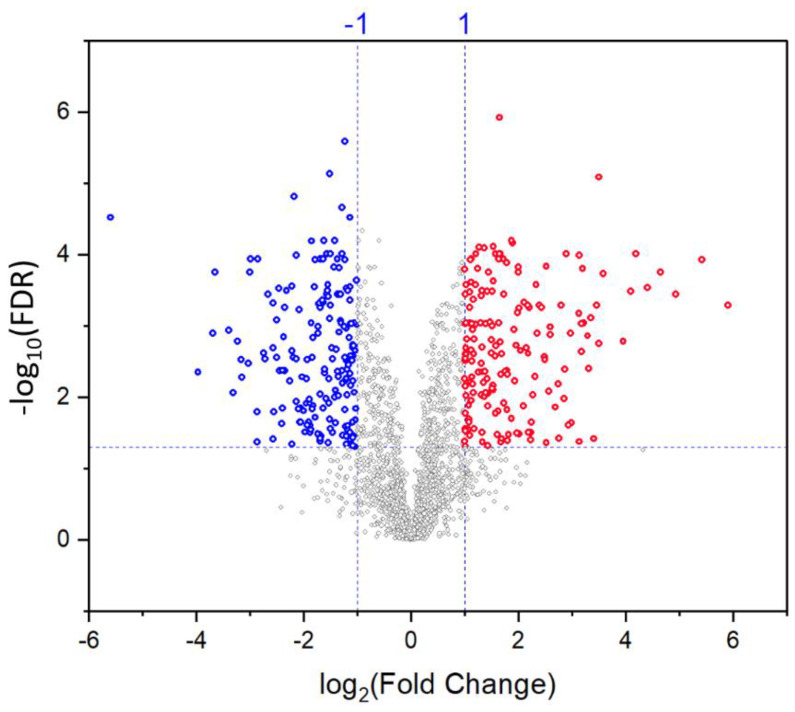
Volcano plot of differentially abundant proteins in the quinoa GCs in response to salt stress. Proteins with decreased and increased abundances (fold change >2, FDR < 0.05) are illustrated as blue and red dots, respectively. Proteins with non-significant expressions and/or fold change of less than 2 are shown as grey dots.

To identify the differentially expressed proteins under saline conditions, a *t*-test using a false discovery rate FDR adjusted *p*-value < 0.05 was performed and the data were analyzed in terms of fold-change relative to unstressed control (ratio values of treated/control). Proteins were considered differentially expressed between control and salt stress conditions if they met two criteria: the statistical criterion (FDR < 0.05) and the biological measure (fold-change >2). Statistical significance of changes and deviations from control could be observed in a volcano plot (Figure 3). Based on these two measures, 387 proteins were differentially abundant under salt stress (185 upregulated and 202 downregulated).

**Figure 4:**
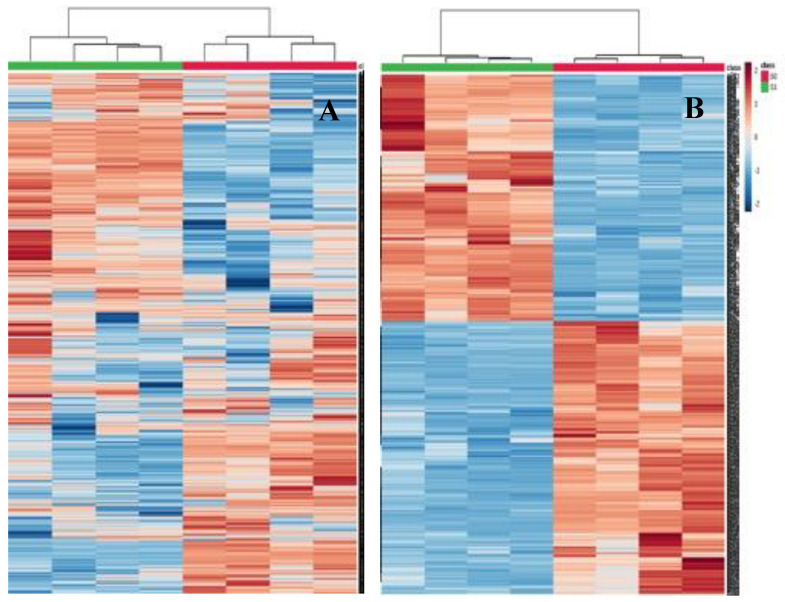
Heat maps based on Z-scores of protein abundance measurements demonstrating abundance patterns of individual proteins in the whole proteome (**A**) and differentially accumulated proteins (**B**). S0 and S1 denote control and saline treatments, respectively.

Heat maps were then used to provide an overview of abundance patterns of individual proteins in the whole proteome data (Figure 4). These heatmaps displayed similarities between biological samples in each treatment.

**Figure 5:**
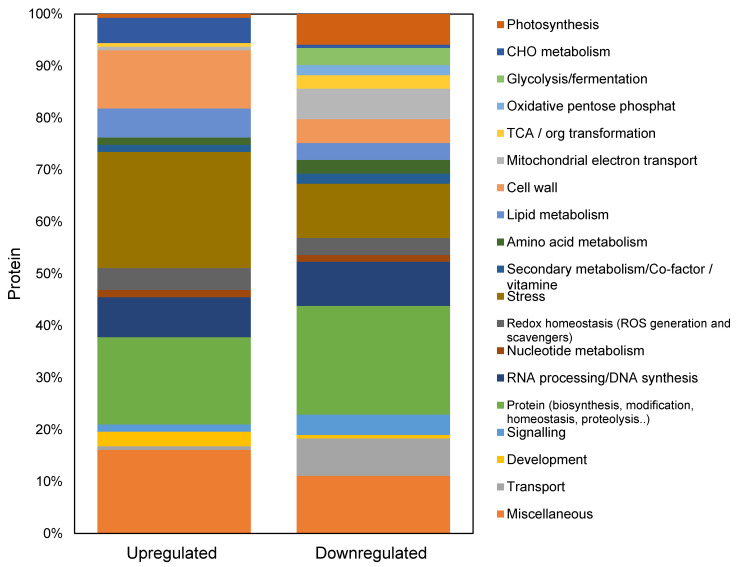
Comparison of differentially accumulated proteins in salt-responsive proteomes of quinoa GC. Upregulated (185 proteins) and downregulated (202 proteins) proteins identified from quinoa GCs have been classified into different categories based on their biological function according to MapMan terms. The Y axes indicate the % of proteins differentially accumulated in the quinoa GCs under 300 mM NaCl. The up and downregulated proteins that were not assigned to a specific functional category were not included.

Sequence-based functional annotations of 185 upregulated and 202 downregulated proteins were then performed using Mercator (Figure 5) to identify possible functions of the differentially abundant proteins. The distribution of protein functions demonstrated that around 20% of proteins were not classified as any functional group. A large number of known functional proteins were classified as the following categories: protein synthesis, protein degradations, post-translational modification, RNA binding, regulation of transcription, DNA (DNA-binding, synthesis and repair), signaling, transport, biotic and abiotic stresses, development, lipid metabolism, cell wall and photosynthesis.

Several proteins involved in general stress responses (including those for osmotic and salt stresses) were found to be upregulated in GCs of quinoa following salinity treatment (Supplemental Table S1), including protein DR29B (50-fold), osmotin-like protein OSML13 (13-fold), polycystin-1, lipoxygenase, alpha-toxin, and triacylglycerol lipase (PLAT) domain-containing protein 3-like (eight-fold), dehydrin early responsive to dehydration (ERD14) (eight-fold), and cationic peroxidase 1 (18-fold). Moreover, antioxidant molecules responsible for cell redox homeostasis such as glutaredoxin-C2- and thioredoxin H-type 1 (TRX) accumulated in salt-treated quinoa GCs. However, downregulation in the expression of the enzymatic antioxidant catalase (CAT) and L-ascorbate peroxidase 5 (APX) was observed in GC. In our study, PER2, PER5, and PER50 were decreased, while PER1 and PER4, PER12 and PER29 were highly increased in quinoa GCs in response to salt stress, suggesting that various members of a protein family might be expressed differently, suggesting potentially different functional roles.

The metabolism of carbohydrates is rapidly modulated in response to environmental stresses. Glucan endo-1,3-beta-d-glucosidase and chitinase are hydrolases, which have been recognized as antifungal proteins, and were highly overexpressed in quinoa GCs under salt conditions (Supplemental Table S1). Glucan endo-1,3-beta-d-glucosidase (increased 27-fold in saline conditions) is a pathogenesis-related (PR) protein, also reported to be associated with salt tolerance and ROS-scavenging in stressed plants [31]. High expression of this protein in GCs, especially in quinoa, may imply the importance of this protein in conferring salt tolerance in halophytes.

Salt stress has been shown to alter patterns of gene expression, potentially via modulation of transcription factors and other proteins, including those involved in alternative splicing. Alternative splicing is a process through which several transcripts and multiple forms of protein are produced from the same gene leading to an increase in proteome diversity. This post translational modification process is induced in plants under various stresses and results in quick adjustment of the function and abundance of key components of stress responses [32]. In this study, splicing factor U2af small subunit B-like is upregulated six-fold in quinoa GCs. This splicing factor, which belongs to a zinc finger CCCH domain-containing protein that is proposed to be important in the salt tolerance of plants, is upregulated six-fold in quinoa GCs [33]. NAP1-related protein X2, which belongs to a family of chaperones and is involved in DNA repair, is also critical under stress conditions [34]. This protein was also upregulated under salt stress (two-fold).

Salinity altered the physical properties of the GC wall. Cell wall-modifying enzymes such as acetyl- and methyl-esterifications of pectin were upregulated in GCs in our study. The acetyl- and methyl-esterifications of pectin are critical for the regulating mechanical properties of the cell wall [35]. Pectin methylesterification is essential for plant responses to environment stresses [36]. For example, PME34 regulates GC flexibility in response to heat stress. Glycine-rich cell wall proteins (GRPs) were upregulated in GCs of quinoa (18-fold). GRPs in the cell wall have 60–70% glycine residues. GRPs generally are involved in the strengthening of biological structural systems or can support the development of expandable organs [37]. Our results are therefore consistent with increased mechanical strength in GCs exposed to salt stress.

### 2.5. Proteins with a Role in Stomatal Movement

Thirty-five proteins were found in this GC proteome study that have a role in stomatal movement (Table 4). For instance, accumulation of ABA receptors and PP2Cs proteins in the ABA signaling pathway can be found in the GC proteome. However, ABA receptor (PYL) showed no differential abundance in GCs of quinoa in response to salt stress while OST1 in GC was only marginally upregulated by salt stress (1.4-fold increase).

### 2.6. Protein Involved in Response to ABA in GC Proteome

**Figure 6:**
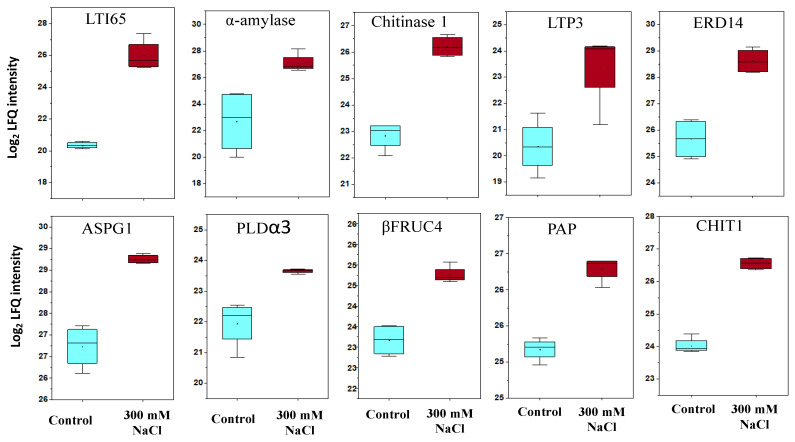
Proteins involved in ABA response, LTI65-low-temperature-induced 65 kDa protein, α-amylase—alpha-amylase, chitinase 1, LTP3—non-specific lipid-transfer protein 3, EDR14—dehydrin, ASPG1—protein aspartic protease in guard cell 1-like, PLD α 3—phospholipase D, Βfruc4—acid beta-fructofuranosidase-like, PAP—plastid-lipid-associated protein, CHIT1—chitinase 1.

The ABA levels in plant tissue tend to elevate with exposure to salt or osmotic stress, indicating the contribution of ABA in stress signal transduction. In this study, ten proteins involved in response to ABA were found to be upregulated in quinoa GC (Figure 6) including LTI65 (50-fold), ASPG1 (4.1-fold), PLD (3.3-fold), α enzyme (21-fold), EDR14 (7.8-fold), LTP3 (8.1-fold), chitinase 1 (10.4-fold), Chit1 (5.8-fold), ΒFRUC4 (three-fold), PAP (22.2-fold).

### 2.7. The Effect of Sucrose, Tryptophan and L-Methionine on Stomatal Conductance

**Figure 7:**
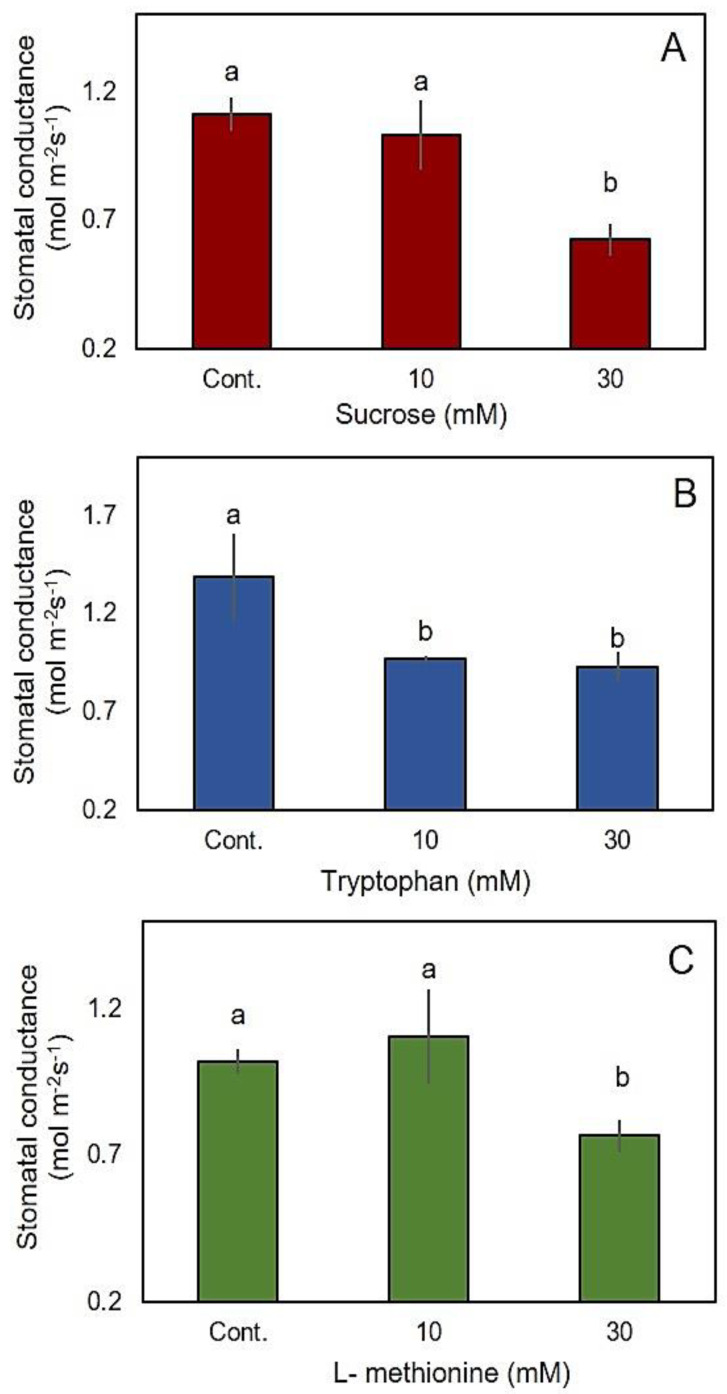
The effect of sucrose (**A**), tryptophan (**B**) and L-methionine (**C**) on stomatal conductance. Mean ± SE (*n* = 5). Data labelled with different lower-case letters are significantly different at *p* < 0.05 based on Tukey’s test.

In our study, sucrose synthase and two proteins related to biosynthesis of tryptophan and 5-methyltetrahydropteroyltriglutamate (involved in L-methionine biosynthesis) were accumulated in the GCs in response to salinity. To understand the physiological rationale behind this phenomenon, we studied effects of exogenous application of sucrose, tryptophan, and methionine on stomatal conductance (Figure 7). The results showed that application of sucrose, tryptophan, and L-methionine was associated with significant reduction in stomatal conductance. Stomatal conductance values were 1.10, 1.39 and 1.02 mol m^−2^ s^−1^ at the control conditions, which decreased to 0.62, 0.93 and 0.77 mol m^−2^ s^−1^ with the application of 30 mM sucrose, tryptophan, and methionine, respectively (Figure 7).

### 2.8. The Effect of Salinity and Pepstatin A on Quinoa Growth and Stomatal Traits

**Figure 8:**
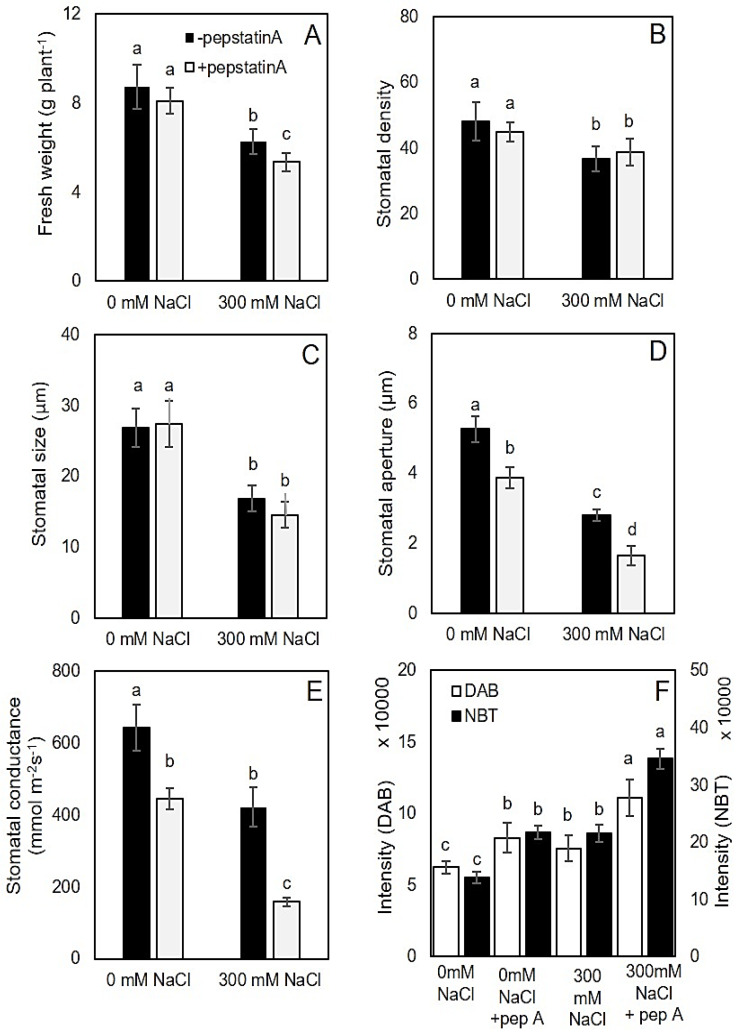
The effects of salinity stress and pepstatin A on different quinoa characteristics. (**A**) Fresh weight; (**B**) stomatal density; (**C**) stomatal size; (**D**) stomatal aperture; (**E**) stomatal conductance; (**F**) intensity of 3,3’-diaminobenzidine (DAB) and NBT staining in the GC as indicator of hydrogen peroxide and superoxide radical production. Data labelled with different lower-case letters (**a**–**d**) are significantly different at *p* < 0.05 based on Tukey’s test.

To understand the functionality of the aspartic proteinases, we blocked their action by spraying pepstatin A on the quinoa leaf for seven subsequent days in control and salt-treated plants. Growth and stomatal trait responses of quinoa plants to salinity stress and pepstatin A are shown in Figure 8A–F. The fresh weight of salt-treated plants was reduced by 28% compared to untreated plants after three weeks of salt stress (Figure 8A). Stomatal density and size (length) in salt-grown plants was reduced by 23% and 37% compared to control conditions, respectively (Figure 8B,C). The impact of pepstatin A on stomatal density and size was statistically insignificant. Application of 2 µM pepstatin A on leaf for a week did not affect the fresh weight of plants grown under control conditions while pepstatin A reduced the fresh weight of salt-grown plants by 14% (Figure 8A).

The stomatal aperture was lower in salt-treated plants than in control counterparts (Figure 8D). It declined by 47% and 58% in untreated and treated plants by pepstatin A, respectively (significant at *p* < 0.05). These decreases in stomatal aperture were accompanied by 34% and 64% decreases in stomatal conductance, compared to control plants (Figure 8E). 

**Figure 9:**
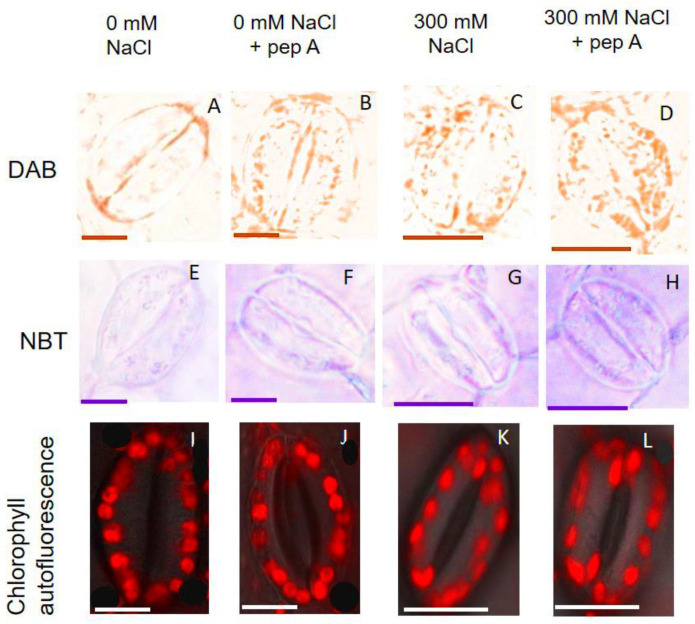
Hydrogen peroxide and superoxide radical generation and chlorophyll autofluorescence in GCs of quinoa in response to salt stress and pepstatin A. (**A**–**D**): 3,3′-Diaminobenzidine (DAB) staining to detect H_2_O_2_ in GCs. (**E**–**H**) Nitro blue tetrazolium (NBT) staining to detect superoxide radicals in GCs. Chlorophyll autofluorescence in GCs (**I**–**L**) was visualized using a laser scanning confocal microscope (LEICA SMD FLCS) at an excitation wavelength of 488 nm and chlorophyll autofluorescence was detected between 629 nm and 697 nm. Bar = 10 µm.

The accumulation of superoxide radical (O^2−^) and hydrogen peroxide (H_2_O_2_) was analyzed using 3,3′-diaminobenzidine (DAB) and nitro blue tetrazolium (NBT) staining, respectively, in quinoa GCs. Plants grown under salinity stress were enriched in O^2−^ and H_2_O_2_. Application of pepstatin A resulted in higher accumulation of O^2−^ and H_2_O_2_ in the GCs and higher ROS accumulation was observed in chloroplast (Figure 8F and Figure 9).

## 3. Discussion

A comparison of differentially abundant proteins in quinoa and sugar beet [23] displayed that only 5% of up and downregulated proteins in quinoa are shared with those in sugar beet. Aspartic protease and non-specific lipid-transfer proteins showed enhanced abundance while catalase and cationic peroxidase showed lower abundances in GCs of both salt-treated plants. Lower levels of catalase may be necessary in the guard cells in saline conditions as H_2_O_2_ functions as a signaling molecule in the guard cell and induces stomatal closure in response to high salinity stress. Moreover, some proteins involved in mitigation of oxidative stress such as L-ascorbate oxidase were presented at elevated levels under non-stress conditions in both species, suggesting that the constitutive accumulation of those proteins in the guard cells of the halophyte quinoa and the salt-tolerant sugar beet can confer augmented tolerance to GCs of both species when exposed to salt stress. The salinity treatment in quinoa exhibited the larger number of differentially abundant proteins compared with sugar beet, suggesting their possible mechanistic role to confer salt-tolerance in quinoa. When differentially abundant proteins in salt-responsive proteomes of guard cells of quinoa and sugar beet were compared based on their biological function, the largest single group of proteins present in both species were those involved in protein biogenesis and disposal (e.g., ribosomal subunits, molecular chaperones, and proteasomal subunits) and the next most abundant protein categories were identified as “stress”. However, the top proteins according to overall abundance in sugar beet and quinoa GCs were dissimilar and they were different from what is previously reported in the other species such as Arabidopsis [19].

### 3.1. Most Abundant Proteins in the GCs Isolated from Quinoa

Rubisco activase was the most abundant protein in quinoa GCs. This is not surprising as Rubisco activase in some halophytes such as *Suaeda salsa* and *Eutrema halophila* is present at higher amounts compared to glycophytes [38]. Furthermore, it has been reported that the promotor region of the Rubisco activase gene is enriched in stress responsive elements including two for temperature, three for dehydration and five for light responses, which means Rubisco activase is a light- and stress-regulated gene [24].

In addition to Rubisco activase, a GDSL motif containing esterases/lipases was found to be among the most highly abundant proteins in quinoa GCs. Previous studies have found that the degree of stomatal aperture in response to low CO_2_ corresponds to increased levels of GDSL esterase/lipases [21]. Furthermore, there is some evidence to suggest involvement of this protein in the salt tolerance mechanism, as a gene belonging to the GDSL-motif lipase family was found to confer enhanced salt and pathogen resistance in Arabidopsis [39].

ABP19 was also an abundant protein in quinoa GCs. ABP19 is a receptor for a hormone auxin that is involved in stomatal patterning and development [40]. Auxin has been proposed to be involved in the modulation of K^+^ channel in the GC [41]. In *Paphiopedilum tonsum*, ABP decreased GC cytoplasmic pH and induced stomatal opening [42].

### 3.2. Photosynthesis in the GCs

Analysis of GC proteins demonstrated that photosynthesis was among the top 10 GO terms in the GC proteome (Supplemental Table S1). Photosynthesis in GCs has been a controversial topic for many years. Although Rubisco and other enzymes for carbon reduction are both present and active in GCs, and the emerging consensus is that photosynthesis does takes place in GCs; however, the role of GC photosynthesis in relation to stomatal behavior is still unclear. A recent experiment on transgenic Arabidopsis [43] with targeted chlorophyll deficiency in GCs has found that GCs are thin and flaccid suggesting that photosynthesis is essential for GC turgor. Another recent study [44] has demonstrated that photosynthesis in GC is necessary for ABA signaling. Photosynthetic electron transport in GCs generates ROS, which act as signaling molecules in stomatal closure induced by ABA.

### 3.3. Stress and Defence-Related Proteins

The high upregulation of dehydrins (LTI65 and ERD14) in salt-treated quinoa GCs implies dehydrin proteins play a role in stomata of halophytes such as quinoa under saline conditions. Early responsive to dehydration (ERD14) belongs to the dehydrin family of proteins containing highly hydrophilic and charged residues that allow a highly flexible structure. They contribute to multiple functions as protecting macromolecular and stabilizing proteins and are the best representative of late embryogenesis abundant (LEA) proteins [45]. In addition to functioning as a chaperone protein, they also act as osmoticum being able to attract water towards the cell to adjust osmotic potential and maintain water status [46].

Polycystin-1, lipoxygenase, alpha-toxin, and triacylglycerol lipase (PLAT) domain-containing protein is generally expressed in vascular tissue and GCs. The levels of this protein increased under salt and ABA stresses [47]. This protein binds to bZIP transcription factors AREB/ABFs that mediate the ABA signaling pathway [47]. ASPG1, a GC specific gene that encodes aspartic protease, was found to increase four-fold in response to salinity. This protein is highly expressed in tolerant genotypes but highly repressed in sensitive ones, indicating a potential role in osmotic stress regulation by osmotic adjustment [48].

In this study, ten proteins involved in response to ABA were found to be upregulated in quinoa GC (Figure 6). Among them, LTI65 is involved in the abscisic acid-activated signaling pathway, as the promoter region of this gene contains two ABA-responsive elements (ABREs).

PLD α enzyme is activated by ABA and produces phosphatidic acid (a signaling molecule), which induces stomatal closure [49]. Plastid-lipid-associated proteins that are lipid-binding physically bind to ABI2 as a key regulator of ABA-mediated response. Overexpression of protein aspartic protease in GCs has been associated with an increase in ABA sensitivity in the stomatal closure [50].

### 3.4. Sucrose, Tryptophan and L-Methionine Induced Stomatal Closure

In the current study, many proteins related to the sucrose/starch metabolism pathway were upregulated in the GC in response to salt stress in quinoa (Supplemental Table S1). The abundance of alpha-amylase1 that is involved in starch breakdown increased by 21-fold under salt stress.

Regulation of stomatal movements by apoplastic sucrose has been a matter of debate [51]. Early studies proposed that sucrose act as an osmolyte that can induce stomatal opening [52]. However, recent studies using functional, physiological, and molecular evidence have proven that sugars including sucrose within the GCs stimulate stomatal closure through involvement of hexokinase [51,53,54]. Hexokinases serve as a sugar-sensing enzyme in plants and overexpression of this gene has been accompanied with enhanced sugar sensing effects, such as a lower photosynthesis rate, and energy supply. In the GCs, overexpression of this gene in the presence of externally added sucrose resulted in significantly lower stomatal aperture in tomato and Arabidopsis [53]. It was shown that hexokinase can initiate an abscisic acid stomatal closure pathway in the GCs [51]. Our proteomics data showed that degradation of starch and production of sucrose have been increased in the GCs in response to 300 mM NaCl. In our recent work, quinoa has displayed a reduced photosynthesis rate under 300 mM salt stress [55], suggesting that sucrose generation in GCs under salt stress is to coordinate photosynthesis with transpiration and reduce water loss.

In our proteomic study, two proteins related to biosynthesis of tryptophan and 5-methyltetrahydropteroyltriglutamate (involved in L-methionine biosynthesis) were accumulated in the GCs by salinity. Accumulation of some amino acids as compatible solute for supporting the balance of water potential in cytosol with vacuole and apoplast in response to salt stress has been reported [56]. In our study, the application of tryptophan and L-methionine was associated with reduced stomatal aperture and conductance, suggesting the involvement of other pathways such as the modulation of channels by amino acids. In an earlier study on barley root under saline conditions, application of amino acids resulted in mitigation of the NaCl-induced potassium efflux for most amino acids including tryptophan and methionine [57]. In contrast, in GCs, exogenous tryptophan, but not methionine, increased K^+^ efflux leading to stomatal closure in *Vicia faba* [58] while L-methionine modulates Ca^2+^ channels to regulate stomatal aperture. It has been suggested that L-methionine activates the calcium channels in GCs, leading to elevation of cytosolic calcium and generation of reactive oxygen species, and stomatal closure [59].

### 3.5. Aspartic Proteinases Are Important in Mitigating Oxidative Stress of GCs

In this study, eight proteins from the aspartic proteinase family were highly upregulated by salt in the quinoa GCs, including four aspartic proteinase CDR1-like proteins (10.3, 11.3, 18.0 and 22.7-fold) and three aspartic proteinase A1-like (2.4, 10.4 and 10.5- fold). Aspartic proteinase in guard cell-1 encoded by a guard-cell specific gene (ASPG1 gene) was also accumulated 4.1-fold in GCs of salt-treated quinoa plants compared to control.

Aspartic proteinases are distributed throughout the plant kingdom and have been implicated in many biological processes such as protein degradation, protein processing, stress responses, and programmed cell death [60,61]. For example, the overexpression of the ASPG1 gene has been reported to be involved in the drought avoidance through the ABA signal transduction pathway in Arabidopsis [50]. VlAP17 is also another aspartic protease gene that is upregulated in Arabidopsis leaves in response to osmotic stress [62].

In our study, the aspartic proteinases that were upregulated in response to salt stress in GCs belong to the pepsin family whose activities have been proven to be inhibited by pepstatin A [63,64]. The results of the blockage of aspartic proteinases by pepstatin A showed that stomatal conductance and aperture were dramatically reduced in pepstatin A-treated plants for both control and salt-stressed plants. Oxidative status analysis of GCs indicated that a higher level of reactive oxygen species was accumulated in the GCs when the activity of the aspartic proteinases was inhibited by pepstatin A. These results indicate that the aspartic protease activity is required for the mitigating salt-induced ROS production in the GCs in salt-affected plants. In Arabidopsis, aspartic proteinases have been suggested to play a role in protecting the integrity of plasma membrane by increasing levels of antioxidants compounds [62]. Overexpression of aspartic proteinase genes enhanced ABA sensitivity in GCs, and resulted in elevated adaptive drought resistance in Arabidopsis [50].

It has been proposed that ROS function as signaling molecules to promote stomatal closure in response to various biotic and abiotic stress conditions [65]. In our previous study on sugar beet GCs [23], a higher concentration of H_2_O_2_ was detected in the GCs under saline conditions compared to control, which was mitigated by ascorbic acid and resulted in higher stomatal conductance and slower response of stomata to dark conditions. In the present study, inhibition of aspartic proteinases impaired stomatal opening due to excessive accumulation of oxidant molecules in the GCs. Aspartic proteinases may control GC ROS homeostasis and allow stomatal opening.

Aspartic proteinases and many proteins whose accumulations have been altered in response to salinity stress in the GCs might be a source of novel candidates with critical roles in salinity tolerance and could be a target for further experiments. Genome sequences of quinoa have been recently published [66,67], which can effectively help in identifying genes related to salinity stress tolerance in this species.

## 4. Materials and Methods

### 4.1. Growth Conditions and GC Preparation

Six seeds of quinoa were planted in a temperature-controlled glasshouse (22 °C, 70% relative humidity, and 12/12 h day/night) at the University of Tasmania. Plants were grown in 8 inch diameter pots filled with potting mix containing 90% composted pine bark; 5% coarse sand; 5% coco peat; gypsum (1 kg·m^−3^); dolomite (6 kg/m^3^); ferrous sulphate (1.5 kg·m^−3^); Osmoform Pre-Mix (1.25 kg·m^−3^) and slow-released fertilizer, Scotts Pro (3 kg·m^−3^). Salt stress was imposed 3 weeks after planting by adding 300 mM NaCl to irrigation water over a period of 3 weeks. GC-enriched epidermal peels were prepared as it was previously described [23,68]. Briefly, fully expanded leaves of well-watered 3–4 week-old quinoa were grinded in a Grindomix blender with a basic solution and crushed ice, then it was passed through a nylon mesh and rinsed with ice-cold distilled water. The entire process was repeated four times. Isolated GCs-enriched fragments were first examined under the microscope to confirm that no contamination of mesophyll fragments or vascular particles were present. The samples were snap-froze in liquid nitrogen and were kept at −80 °C until they were used for protein extraction.

### 4.2. Label-Free Quantitative (LFQ) Proteomic Analysis of Quinoa GCs:

Protein samples from four biological replicates per treatment were extracted essentially as published elsewhere [23]. Following homogenization by grinding in liquid nitrogen, proteins were precipitated using TCA-acetone containing 0.07% (*v*/*v*) 2-mercaptoethanol, followed by two acetone washes and resuspension in a denaturation buffer (7 M urea etc). Protein concentrations were estimated using the Pierce 660 nm spectrophotometric assay. Following reduction and alkylation by the standard methods, protein digests (30 μg/sample) were prepared using the SP3 method for sample clean-up and digestion with 1.2 μg MS grade trypsin/LysC [69]. Peptide samples of about ~1 μg were analyzed by LC/MS using an Ultimate 3000 RSLCnano and Q-Exactive HF (Thermo Scientific) as described earlier [23]. Raw DDA-MS) files were analyzed using the MaxQuant platform for LFQ proteomics (version 1.6.5.0) utilizing the Andromeda search engine to match MS/MS spectra against the NCBI *Chenopodium quinoa* (wild) proteome database (63,475 entries downloaded on 20/10/18). Search parameters were set to default values for Orbitrap mass spectrometry using a 1% FDR for both peptide-spectrum matches and protein identification. Protein groups identified either as potential contaminants (prefixed with CON_), identified by modified site only, by reverse database matching or on the basis of a single matching peptide were removed. 

### 4.3. Determination of Relative Protein Abundance and Statistical Analysis

We utilized MaxLFQ, the MaxQuant algorithm for peptide intensity determination and normalization, using pair-wise comparison of unique and razor peptide intensities and a minimum ratio count of 2. The protein group output files generated by MaxQuant analysis were processed as follows: the normalized label-free quantification (LFQ) intensity values, MS/MS counts and the numbers of razor and unique peptides for each of the identified proteins were imported into Perseus software version 1.5.031 (http://perseus-framework.org/). Protein groups identified either as potential contaminants (prefixed with CON_), identified by modified site only, by reverse database matching or on the basis of a single matching peptide were removed. LFQ intensity values were then log2–transformed and then a filter was applied to include only proteins detected in a minimum of 70% of the samples. Missing values were replaced with random intensity values for low-abundance proteins based on a normal distribution of protein abundances using default MaxQuant parameters.

### 4.4. Stomatal Conductance in Plants Treated with Sucrose, Tryptophan, and L-Methionine

The effects of 10 and 30 mM exogenously applied sucrose tryptophan and L-methionine on stomata aperture were studied by spraying the above agents on the leaf and measuring stomatal conductance two hours later under normal light in the glasshouse using a Li-Cor 6400 gas analyzer system (Lincoln, NE, USA).

### 4.5. Exogenous Application of Pepstatin A

Quinoa plants were grown for 3 weeks under 300 mM salt and control conditions. Pepstatin A was dissolved in 100% ethanol and was applied to leaves at a final concentration of 2 mM. After a week, fresh weight and stomatal parameter intensity of DAB and NBT staining in the GCs as an indicator of hydrogen peroxide and superoxide radical production were then determined. A SC-1 leaf porometer was used for stomatal conductance in control and pepstatin A-treated plants.

### 4.6. In Situ Detection of O_2_^−^ and H_2_O_2_ in GCs and Imaging

Hydrogen peroxide in GCs was detected using 3,3′-diaminobenzidine (DAB) staining. For this purpose, leaves from control and salt-treated plants were incubated in DAB solution (1 mg·mL^−1^ DAB, and 50 mM Tris-acetate buffer (pH = 5)). Vacuum-infiltrating was applied for 10 min in the dark. Then, the samples were kept on the shaker overnight at 75 rpm shaking speed. Superoxide radical (O^2−^) was detected using nitro blue tetrazolium (NBT). The leaf samples were incubated in 0.1 mg mL^−1^ NBT solution prepared in 25 mM HEPES buffer (pH = 7.6) for four hours. The stained samples were then de-stained using an ethanol:glycerol:acetic acid (3:1:1) solution two times before visualizing in light microscopy. The washing process for DAB staining and NBT staining was carried out under 90 °C and 65 °C, respectively. Chlorophyll autofluorescence was visualized using a laser scanning confocal microscope (LEICA SMD FLCS) at an excitation wavelength of 488 nm and chlorophyll autofluorescence was detected between 629 nm and 697 nm.

### 4.7. Statistical Analyses

Data were analyzed using IBM SPSS Statistics software, version 27 (IBM Corp., Armonk, NY, USA). Statistical significance was determined by one-way ANOVA analysis based on Tukey’s test. The differences between means were considered statistically significant as P-values were less than 0.05.

## 5. Conclusions

This study demonstrated that salinity stress significantly altered the protein profile of quinoa GCs where the abundance of many proteins including signaling molecules, enzyme modulators, transcription factors and oxidoreductases was changed. Furthermore, many proteins involved in osmotic or salinity stress as well as in response to ABA were found to be highly abundant or upregulated in quinoa GC following salinity treatment. Additionally, exogenous application of sucrose and amino acids (tryptophan and L-methionine) resulted in reduced stomatal aperture and conductance, suggesting that it could be advantageous for plant adaptation to salt stress. Inhibition of aspartic proteinases impaired stomatal opening due to excessive accumulation of ROS in the GCs, suggesting the important role of aspartic proteinases in GC ROS homeostasis and stomata movements.

## Figures and Tables

**Table 1:** Top eight gene ontology (GO) terms of quinoa guard cell (GC) proteome.

GO Term	Term	Query Item	FDR *
GO:0006412	translation	122	2.4 × 10^−22^
GO:0008152	metabolic process	854	3.1 × 10^−18^
GO:0006091	generation of precursor metabolites and energy	46	4.5 × 10^−15^
GO:0015979	photosynthesis	39	3.9 × 10^−12^
GO:0005975	carbohydrate metabolic process	115	4.4 × 10^−12^
GO:0009056	catabolic process	59	4.4 × 10^−10^
GO:0009058	biosynthetic process	253	9.6 × 10^−7^
GO:0006950	response to stress	76	1.8 × 10^−4^

* False Discovery Rate

**Table 2:** A representative selection of the signaling proteins that were identified in quinoa GC proteome (the complete list of signaling proteins is provided in Supplemental Table S1).

Protein Code	Name	Signal Type
XP_021772273	mitochondrial proton/calcium exchanger protein-like	Calcium
XP_021720398	Calcium-binding EF-hand family protein	Calcium
XP_021730224	probable calcium-binding protein CML13	Calcium
XP_021743759	calcium-binding allergen Ole e 8-like	Calcium
XP_021724195	calcium-binding protein CML49	Calcium
XP_021740220	cryptochrome-1-like isoform X3	Calcium
XP_021761282	calnexin homolog	Calcium
XP_021742145	serine/threonine protein phosphatase 2A	Calcium
XP_021751211	14-3-3-like protein	14.3.3
XP_021775698	14-3-3-like protein D	14.3.3
XP_021772761	GTP-binding protein SAR1A	G-proteins
XP_021772956	mitochondrial Rho GTPase 1	G-proteins
XP_021760299	dynamin-related protein 5A	G-proteins
XP_021768021	ras-related protein RABD2c-like	G-proteins
XP_021763216	guanylate-binding protein 2-like	G-proteins
XP_021738598	ras-related protein Rab7-like	G-proteins
XP_021731959	nuclear pore complex protein NUP50A-like	G-proteins
XP_021765995	guanylate-binding protein 3-like	G-proteins
XP_021736812	nuclear pore complex protein NUP50A-like	G-proteins
XP_021735409	nucleolin 1-like	G-proteins
XP_021748152	RAN GTPase-activating protein 2	G-proteins
XP_021736812	nuclear pore complex protein NUP50A	G-proteins
XP_021735409	nucleolin 1-like	G-proteins
XP_021748152	RAN GTPase-activating protein 2	G-proteins
XP_021751681	G- nucleotide diphosphate dissociation inhibitor	G-proteins
XP_021716254	phototropin-1-like isoform X1	Light
XP_021740244	protein EXORDIUM-like	Light
XP_021774295	phytochrome B-like	Light
XP_021750479	NAD(P)-binding Rossmann-fold superfamily	Light
XP_021738894	COP9 signalosome complex subunit 5a-like	Light
XP_021714332	PLC-like phosphodiesterases superfamily	MAP kinases
XP_021774801	mitogen-activated protein kinase MMK1	MAP kinases
XP_021754221	mitogen-activated protein kinase MMK2	MAP kinases
XP_021723308	Leucine-rich repeat protein kinase family protein	RK. LRR III
XP_021746237	LRR receptor-like ser/thre-protein kinase	RK. LRR VI
XP_021749023	inactive LLR receptor-like protein kinase	RK. LRR VII
XP_021775620	leucine-rich repeat receptor-like protein kinase	RK. LRR VII
XP_021718843	leucine-rich repeat receptor-like protein kinase	RK. LRR VII
XP_021772032	DNA damage-repair/toleration protein DRT100	RK. LRR XI

**Table 3:** A representative selection of the transporter proteins that were identified in quinoa GC proteome. The full list of transporter proteins is given in Supplemental Table S1.

Accession No.	Name	Transporter
XP_021715294	plasma membrane-associated cation-binding protein	cation
XP_021762724	ABC transporter F family member 4-like	ABC
XP_021743351	ABC transporter C family member 2-like	ABC
XP_021776035	ABC transporter G family member 22-like	ABC
XP_021766195	ABC transporter I family member 19-like	ABC
XP_021720939	calcium-transporting ATPase 4	calcium
XP_021751211	calcium-transporting ATPase 10	calcium
XP_021744756	bifunctional monothiol glutaredoxin-S16	calcium
XP_021760697	pyrophosphate-energized vacuolar membrane H^+^ pump	H^+^ pump
XP_021769151	probable aquaporin PIP1-4	PIP
XP_021765658	mitochondrial carnitine/acylcarnitine carrier-like protein	metabolite
XP_021738293	mitochondrial dicarboxylate/tricarboxylate transporter DTC	metabolite
XP_021756032	mitochondrial phosphate carrier protein 3, mitochondrial	metabolite
XP_021757591	cation/H^+^ antiporter 18-like	cation
XP_021736780	plastidic ATP/ADP-transporter-like	Misc
XP_021738681	V-type proton ATPase subunit a3-like	ATPases
XP_021730105	V-type proton ATPase subunit C-like	ATPases
XP_021761683	ATPase 11, plasma membrane-type-like	ATPases
XP_021765334	V-type proton ATPase subunit G 1-like	ATPases
XP_021739675	V-type proton ATPase catalytic subunit A	ATPases
XP_021732700	V-type proton ATPase catalytic subunit A-like	ATPases
XP_021738896	plasma membrane ATPase 4-like	ATPases
XP_021762284	V-type proton ATPase subunit d2	ATPases
XP_021765533	V-type proton ATPase subunit B 2	ATPases
XP_021754298	V-type proton ATPase subunit E-like	ATPases
XP_021772280	V-type proton ATPase subunit H-like isoform X2	ATPases
XP_021714458	mitochondrial outer membrane protein porin 2-like	Porin
XP_021761841	mitochondrial import receptor subunit TOM40-1-like	Porin
XP_021758463	mitochondrial outer membrane protein porin of 34 kDa	Porin
XP_021717525	K^+^ efflux antiporter 2, chloroplastic-like	Potassium
XP_021762166	probable voltage-gated potassium channel subunit beta	Potassium
XP_021753247	monosaccharide-sensing protein 2-like	Sugar
XP_021726328	sugar carrier protein C-like	Sugar
XP_021757156	plastidic glucose transporter 4-like	Sugar
XP_021760460	sucrose transport protein-like isoform X1	Sucrose
XP_021752898	chloride channel protein CLC-b-like	anions
XP_021739774	ATPase ASNA1 homolog	anions
XP_021772050	ADP, ATP carrier protein 1, mitochondrial-like	cation

**Table 4:** Proteins with a direct role in GC function.

Accession No	Name	Fold Change NaCl/Control
XP_02174988	abscisic acid receptor PYL2	1.1
XP_021736717	phospholipase D alpha 1-like	3.3 *
XP_021739586	GDPDL3-like	2.1 *
XP_021760770	ricin B-like lectin EULS3	2.0 *
XP_021754221	MMK2-like	1.6 *
XP_021759066	GDPDL3-like	1.5 *
XP_021738483	OST1	1.3 *
XP_021723531	hexokinase-1-like	0.9 *
XP_021715572	clathrin heavy chain 1-like	0.8 *
XP_021723433	plasma membrane ATPase 4-like	0.5 *
XP_021761237	calcium sensing receptor, chloroplastic-like	0.5 *
XP_021734330	protein flowering locus t-like	0.3 *
XP_021776446	protein thylakoid formation1	0.3 *
XP_021775775	uncharacterized protein LOC110739633	0.2 *
XP_021776385	protein phosphatase 2C	0.4
XP_021714641	DNA-directed RNA polymerases II, IV and V	2.5
XP_021721800	BLUS1-like	2.2
XP_021757551	MMK2-like	1.9
XP_021723484	serine/threonine-protein kinase STY8-like	1.8
XP_021760806	carbonic anhydrase, chloroplastic	1.7
XP_021740220	cryptochrome-1-like isoform X3	1.4
XP_021753299	phototropin-2-like	1.3
XP_021731588	glycine-rich RNA-binding, ABA-inducible protein	1.1
XP_021774295	phytochrome B-like	1.1
XP_021742577	translationally-controlled tumor protein homolog	1.1
XP_021716254	phototropin-1-like isoform X1	1.0
XP_021741723	carbonic anhydrase, chloroplastic-like	1.0
XP_021774801	MMK1-like	0.9
XP_021760799	phospholipase D alpha 1-like	0.9
XP_021758723	chlorophyll a-b binding protein 3, chloroplastic	0.7
XP_021745518	plasma membrane-associated cation-binding protein 1	0.7
XP_021731589	glycine-rich RNA-binding protein-like	0.7
XP_021723344	phosphoglycerate mutase-like	0.6
XP_021760744	vesicle-associated membrane protein 711	0.6
XP_021769207	clathrin heavy chain 1-like	0.5

* denotes a significant protein difference between control and salt stress (Student’s *t*-test, *p* < 0.05; fold change >2.0).

## Data Availability

Not applicable.

## Supplementary Materials

Supplementary Materials can be found at https://www.mdpi.com/1422-0067/22/1/428/s1.

## Abbreviations

ABA	abscisic acid
DAB	3,3′-diaminobenzidine
FDR	False Discovery Rate
GC	guard cells
KEGG	Kyoto Encyclopedia of Genes and Genomes
NBT	nitro blue tetrazolium
NCBI	National Center for Biotechnology Information
ROS	Reactive oxygen species
